# Surgical Resection of Native Viscera to Manage Persistent Ascites after Multivisceral Transplant

**DOI:** 10.1155/2020/8863508

**Published:** 2020-10-07

**Authors:** Brian I. Shaw, Andrew S. Barbas, Debra L. Sudan

**Affiliations:** Department of Surgery, Duke University, Durham, NC, USA

## Abstract

Multivisceral transplantation is the therapy of choice in patients with diffuse portomesenteric thrombosis. In the present case, we describe a patient who had persistent ascites after multivisceral transplant. The patient was initially diagnosed with a chyle leak which was cured via embolization. When this did not cure her ascites, reexploration proved the etiology to be at least partially attributable to persistent hypertension in the retained viscera. This was cured with the resection of her native viscera. This case highlights the importance of resection of all congested viscera at the time of transplantation in patients with diffuse portomesenteric thrombosis, the utility of preoperative embolization techniques in assisting this, and also the ability to perform delayed resection if necessary.

## 1. Introduction

Multivisceral transplantation is increasingly common [1]. One indication for multivisceral transplant is diffuse portomesenteric thrombosis. In this scenario, orthotopic liver transplantation (OLT) alone does not ameliorate portal hypertension due to thrombosis of the mesenteric venous drainage and is not technically feasible due to dense thrombus in the portal and superior mesenteric veins precluding portal flow to the liver allograft. A single center experience has shown that multivisceral transplant leads to acceptable outcomes in these patients as long as concurrent kidney transplantation is not required [2, 3].

Here, we report a multivisceral transplant (liver, pancreas, small bowel) for a patient with extensive thrombosis of the native mesenteric vasculature whose postoperative course was complicated by refractory ascites, not ameliorated by either lymphangiographic embolization or shunting procedures. This patient required surgical resection of the organs drained by the native mesenteric venous system (including spleen, stomach, duodenum, and pancreas) for complete resolution of symptoms.

## 2. Case Presentation

The patient was a 45-year-old woman who first presented to our center due to abdominal pain 7 years prior to transplantation. At initial presentation, she had cryptogenic cirrhosis requiring large-volume paracentesis. She had undergone no procedures except paracentesis on presentation to our center. Initial magnetic resonance imagining (MRI) demonstrated possible bowel ischemia as well as thrombosis of her portal, splenic, and superior mesenteric veins. Approximately 190 cm of ischemic small bowel was resected over multiple operations. Liver biopsy at this time showed central lobular ischemic injury and early fibrosis.

She next underwent a transjugular intrahepatic portosystemic shunt (TIPS) procedure for continued ascites after thrombolysis of her portomesenteric clot, which allowed for performance of the procedure. She required multiple TIPS revisions due to thrombosis in spite of systemic anticoagulation. Hypercoagulable workup revealed a weakly positive lupus anticoagulant antibody, heterozygosity for the prothrombin G20210A polymorphism, heterozygosity for the JAK2 V617F mutation, and elevated platelet counts, consistent with essential thrombocytosis.

**Figure 1:**
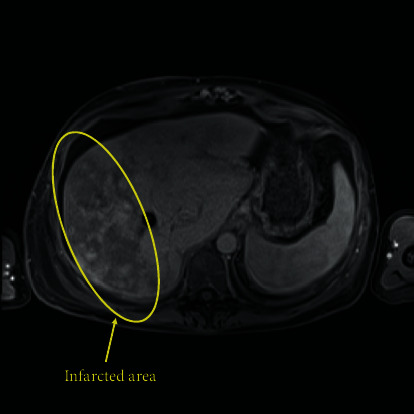
Preoperative MRI demonstrating multifocal infarction of the right lobe of the liver, with TIPs in place.

She was maintained on hydroxyurea for some time before she was admitted to the hospital for TIPS occlusion with ascites. She also had occlusion of her hepatic veins, cavernous transformation of the portal vein, and multifocal intrahepatic and extrahepatic portal venous thromboses and associated regenerative nodules (Figure 1). Due to recurrent TIPS occlusion, she was evaluated for and underwent a combined liver, pancreas small bowel transplant 7 years after her initial presentation. Her calculated MELD-Na at that time was 22. Of note, her only pretransplant comorbidities not related to her hypercoagulable state were prior melanoma and squamous cell carcinoma treated and cured as well as situational migraines and anxiety.

During the transplant, a large volume of chylous ascites was encountered. Due to significant collateral vessels obscuring the planes for dissection around the head of the pancreas and the fact that she had few esophagogastric varices (likely indicative of sufficient venous drainage of the bowel), the decision was made to defer complete removal of the native organs (stomach, pancreas, spleen) draining into the clotted native portomesenteric system.

The multivisceral graft was implanted using a conduit of donor thoracic aorta for inflow, anastomosed to the native supraceliac aorta. Venous drainage was via the vena cava above the level of clot. Ultimately, the recipient jejunum and ileum were resected, and an ileocolostomy with a Santulli (“Chimney”) ileostomy was created. The patient received a total of 4 units of packed red blood cells, 4 units of fresh frozen plasma, and 3 units of platelets.

Postoperatively, the patient experienced difficulty resuming enteral nutrition due to chylous ascites. Therefore, she was discharged on total parenteral nutrition (TPN). Explant pathology of the native liver showed severe parenchymal congestion and nodular regenerative hyperplasia with centrilobular perisinusoidal fibrosis.

**Figure 2:**
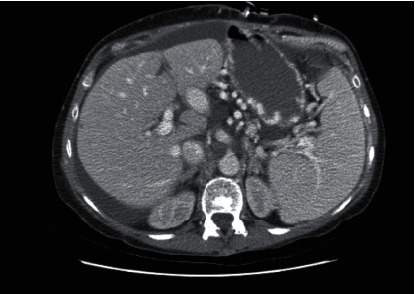
Posttransplant CT scan demonstrating large varices, splenomegaly, and ascites.

**Figure 3:**
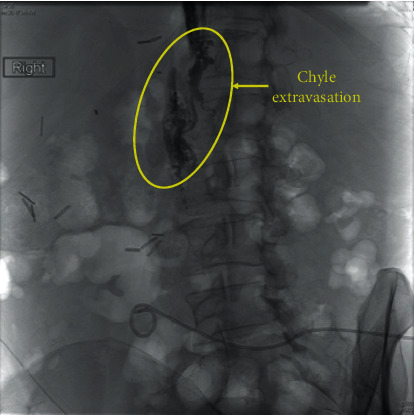
Representative image showing chyle leak interrogated during lymphangiogram. This was embolized but did not significantly improve the patient's symptoms.

Her postoperative course was complicated by multiple readmissions secondary to refractory chylous ascites. Postoperative computed tomography (CT) scan redemonstrated massive splenomegaly and large varices (Figure 2). She underwent Denver shunt placement 4 months after transplant. Though this provided some relief, she required a total of four revisions of her shunt over the course of 1.5 years. Due to the fact that her shunt was only ever patent for a short period of time, lymphangiography was pursued and a chyle leak was identified and embolized (Figure 3). However, the procedure did not appreciably improve her symptoms. She next underwent surgical exploration; however, the ascites at this time was unexpectedly nonchylous and no direct lymphatic leak was identified.

Given the negative exploration and the nonchylous and persistent nature of her ascites, consideration was given to residual portomesenteric hypertension as the potential cause of her ascites. Three years after her initial transplant, the decision was made to proceed with surgical resection of her the native organs draining into the native obstructed portomesenteric system. In preparation for this procedure, her native spleen was embolized. She then underwent explant of her native distal stomach, duodenum, pancreas, and spleen. The native proximal superior mesenteric artery was ligated (supplying the duodenal, pancreatic, and proximal jejunal arterial branches). The common hepatic artery distal to the left gastric artery was also ligated in order to preserve blood supply to the proximal native stomach. Thereafter, mobilization of the markedly enlarged spleen and transection of the mid gastric body allowed for excision of the duodenum, pancreas, (including the retropancreatic thrombosed portal and splenic veins), spleen, distal stomach, proximal jejunum, and a short length of the native transverse colon. Gastrointestinal continuity was restored by the creation of a roux-en-y gastrojejunostomy utilizing the transplanted small bowel and new ileocolostomy.

The patient recovered well from this operation with complete resolution of her now nonchylous ascites and was discharged on postoperative day 16. Since this operation, the patient has been admitted to the hospital only briefly for choledocholithiasis requiring endoscopic retrograde cholangiopancreatogram (ERCP) and an episode of malaise and cramping abdominal pain with diarrhea that improved with conservative management.

## 3. Discussion

This case illustrated a unique clinical scenario where portal hypertension persisted despite liver transplantation. While complete upper GI tract exenteration of the native mesenteric organs at the time of the index multivisceral transplantation has been described [2, 3], this case occurred early in our institutional experience. The consequences of retaining a small portion of native viscera were weighed intraoperatively against the risks of further visceral mobilization in light of her extensive venous collaterals. Our team presumed the intact peritoneal cavity would reabsorb any fluid from a small degree of portal hypertension in the native stomach and duodenum. Following the development of refractory ascites, the initial concern was for chyle leak. After radiologic embolization and resolution of her chyle leak, a decision was ultimately made to resect her native residual stomach, duodenum, pancreas, and spleen to resolve her remaining portal hypertension in the native proximal gastrointestinal tract. Splenic embolization was pursued to facilitate explant of the native viscera by decreasing venous flow in the collaterals formed around the native portal/splenic thrombosed veins. More recently, multiple centers including ours have reported the routine use of preoperative embolization of major mesenteric arterial vasculature (celiac axis and SMA), in order to prevent the clinical scenario experienced in this transplant procedure with difficult dissection due to extensive venous collateral formation [4, 5].

Chylous ascites following multivisceral transplantation has been reported with an incidence as high as 20% [6]. Some transplant centers have reported a lower rate (~7%) with aggressive use of early postoperative TPN to reduce lymphatic leakage and report resolution of posttransplant ascites within a few weeks [7]. The persistence of our patient's chyle leak despite TPN support and initial restriction of oral intake is unusual. Additionally, after embolization of the identified chyle leak, it was not immediately recognized that the persistence of the ascites was not chylous, but secondary to portomesenteric hypertension of the native viscera. The two etiologies for her persistent ascites likely led to a delay in diagnosis.

A Denver shunt was initially utilized in light of successful reports in patients with chylous ascites and due to the expectation that the ascites would resolve with time after transplant [8]. Denver shunts have been mostly described in the management of portal hypertensive ascites [9]. However, as in our patient, the difficulty of this therapy is the low patency rate, as evidenced by the need for multiple revisions [10]. This may be due in part to the composition of the ascites which is drained. For this reason, these devices are mostly reserved for patients with limited life expectancies or short term use, as initially anticipated in this patient.

We present a novel case of a patient with diffuse portomesenteric thrombosis with preoperative ascites who, though she tolerated the initial transplant procedure well, developed refractory postoperative ascites. This was ultimately cured through both embolization of the site of chyle leak and subsequent removal of the proximal native abdominal viscera. This report outlines two lessons learned. First, it suggests that persistent chylous ascites after abdominal organ transplant should be investigated by radiographic examination of the lymphatic system and embolization of leak when identified if low-fat diet and/or TPN fail to resolve the leak. Second, leaving in even small remnants of abdominal viscera with venous occlusion at the time of multivisceral transplantation for portomesenteric occlusion can lead to persistent ascites. In these patients, consideration for preoperative arterial embolism of the native mesenteric vasculature to facilitate complete resection of the affected viscera is advisable. Delayed resection, while not desirable, is feasible.

## Data Availability

Data are available upon request to the corresponding author.

## Abbreviations

CT: Computed tomography
ERCP: Endoscopic retrograde cholangiopancreatogram
MELD-Na: Model for end-stage liver disease-sodium
MRI: Magnetic resonance imaging
OLT: Orthotopic liver transplant
TIPS: Transjugular intrahepatic portosystemic shunt
TPN: Total parenteral nutrition.
